# Factors Associated with Attrition: Analysis of an HIV Clinic in Japan

**DOI:** 10.1007/s10903-020-00982-y

**Published:** 2020-02-18

**Authors:** Chieko Hashiba, Mayumi Imahashi, Junji Imamura, Masashi Nakahata, Ayumi Kogure, Hideto Takahashi, Yoshiyuki Yokomaku

**Affiliations:** 1 Department of Nursing, Nagoya Medical Center, 4-1-1 Sannomaru, Naka-ku, Nagoya, 460-0001 Japan; 2 Department of Infectious Diseases, Nagoya Medical Center, 4-1-1 Sannomaru, Naka-ku, Nagoya, 460-0001 Japan; 3 National Institute of Public Health, 2-3-6 Minami, Wako, 351-0197 Japan; 4 Department of Infectious Diseases, Sendai Medical Center, 2-8-8 Miyagino Miyagino-ku, Sendai, 983-8520 Japan

**Keywords:** Medical interpreter, Language barrier, People living with HIV/AIDS, Health disparity

## Abstract

This study evaluated the impact of a public medical interpreter on the follow-up clinic attendance rate of foreign-born people with HIV who live in Japan. Participants were patients who visited Nagoya Medical Center from 2009 to 2016. Lost to follow-up was defined as an absence from follow-up visits for more than six months without any notification. A log-rank test was conducted to compare the lost-to-follow-up rates by patients’ nation of origin and medical interpreter use. Of the 931 participants, 114 were foreign patients, whose overall attendance rate at 5 years was 75.5%, which was significantly lower than that of Japanese patients (94.1%, *p* < 0.001). There was no significant difference in regular attendance with respect to medical interpreter use (*p* = 0.09). Social support in addition to a medical interpreter may be needed to improve attendance rates in the study population.

**Electronic supplementary material:**

The online version of this article (10.1007/s10903-020-00982-y) contains supplementary material, which is available to authorized users.

## Background

The Japanese healthcare system has made a substantial contribution to promoting individuals’ health in Japan. Nevertheless, people with limited Japanese proficiency, e.g., immigrants, may have limited access to appropriate health information or not receive the full benefits of the healthcare system due to their language barrier [1]. Notably, English is not often used, even in metropolitan cities in Japan. Thus far, no national-level law in Japan regarding the provision of medical interpreting services to patients with limited Japanese proficiency has been established, although some local authorities have introduced the provision of medical interpreters [2].

The difficulties of people living with HIV (PLWH) in Japan who have a language barrier experience difficulties, including connecting with healthcare facilities to maintain adequate drug adherence [3], dealing with public offices to receive health insurance, and finding an appropriate interpreter or one who can rectify the existing life-threatening interpretation errors that occur when ad hoc interpreters are used [4].

To reduce language barriers among PLWH in our clinic, in 2012, we introduced the “Aichi Medical Interpretation System” (AiMIS) [5]. This confidential service delivers medical interpreters to healthcare settings, arranges tele-interpreters, translates medical documents, and provides multilingual manuals for patient care. All medical interpreters in AiMIS are trained and certified before they are dispatched. Patients can choose an interpreter in person or over the phone. When patients request an attending medical interpreter for their appointments, a clinic clerk contacts AiMIS, and the cost of this service is met by the clinic.

Thus, this cross-sectional study was aimed to examine whether the use of a medical interpreter among PLWH with limited Japanese proficiency has a positive association on regular attendance to the HIV clinic.

In this study, we (1) compared the rate of clinic attendance between foreign-born and Japanese patients, (2) compared the attendance rate among foreign-born patients based on AiMIS use, and (3) identified the determinants of regular follow-up visits.

We hypothesized that (1) Japanese patients would have a better attendance rate than would foreign-born patients, (2) AiMIS use would improve the attendance rates of foreign-national PLWH at our HIV clinic, and (3) the longer the foreign nationals live in Japan, the lower the rate of lost to follow-up.

## Methods

### Participants

Participants were recruited from an HIV specialty clinic at Nagoya Medical Center, Nagoya City, Aichi prefecture, and all patients in this clinic were HIV positive. The clinic has approximately 1300 HIV patient visits per year.

The inclusion criteria for study participation were patients who visited our clinic initially between October 2009 and December 2016 and were at least 15 years old. The exclusion criteria were aged less than 15 years old and who had visited our clinic initially before October 2009 or after December 2016. One patient who grew up in Japan and was a native Japanese speaker was classified as a foreign-born participant because his nation of origin was not Japan.

We introduced a service in which we provided a medical interpreter to all the patients whose nation of origin was other than Japan. All medical interpreters were sent based on patient consent.

### Data Collection

We analyzed demographic and clinical data recorded in the electronic medical records of patients first registered in our clinical database between October 2009 and December 2016. The requirement for written informed consent was waived. The opportunity to opt out of this study was made clear via an announcement in the clinic. Nurses in our clinic explained this study and the opportunity to opt out of this study to the patients after their appointments with doctors. For those with an insufficient understanding of Japanese, the nurses explained the study, using medical interpreters or healthcare workers who spoke English fluently.

### Measures

Demographic variables included age, gender, and native language. Age was defined as the age when a patient was first registered in our database. Clinical and socioeconomic factors included years of residence in Japan, sexual orientation, whether living with family, health insurance, employment status, social welfare, disability certificates, treatment of HIV/AIDS, disease status at initial visit, and use of AiMIS. We also collected the number of times that AiMIS was used based on the communication records between AiMIS and our clinic when we requested interpreter services. The frequency of AiMIS use was recorded from May 2012 to August 2017. The lost-to-follow-up period was October 2009 to August 2017.

The primary efficacy analysis of AiMIS involved a comparison of the rate of foreign-born patients who visited our clinic regularly at successive points in time. Lost to follow-up was defined as not visiting our clinic for more than six months after the last visit without any notification. Therefore, we excluded patients from the lost-to-follow-up group if they returned to their home countries and notified our clinic. Treatment failure was defined as having an HIV-1 viral load of more than 200 copies/ml [6].

### Analysis

#### Statistical Analysis

Fisher’s exact test was performed to compare the rates of certain factors (sex, sexual orientation, socioeconomic status at initial visit, AiMIS use, and distance from Nagoya Medical Center) between the lost-to-follow-up and non-lost-to-follow-up groups. To compare continuous variables, we used a Mann–Whitney *U* test, as data on the patients’ age and years of residence in Japan were not normally distributed. A *p-*value of less than 0.05 was considered statistically significant. Data were analyzed with STATA software, version 15.0 (College City, TX, USA). The distance from our clinic was measured by spatial analysis, which is described in the supplemental material.

The Kaplan–Meier method was used to compare the regular visit rate (1) between Japanese and foreign-born patients, (2) between patients who used AiMIS for their appointment and patients who did not, and (3) stratified by the frequency of AiMIS use. The log-rank test was used to examine the differences between the groups.

To analyze patients’ demographics and socioeconomic status as determinants of regular attendance, we used the multivariate statistical model after adjusting for sex, sexual orientation (heterosexual vs. other), socioeconomic factors, AiMIS use, age (> 35 years vs. other), years of residence in Japan (> 10 years vs. other), and distance from the clinic (> 17 km vs. other). We conducted a univariate analysis for each factor and then chose the factors based on a clinical perspective. Finally, we selected a model of multivariate analysis and conducted a multivariate analysis.

The Institutional Review Board of Nagoya Medical Center approved the protocol and use of clinical data for this study (#2017-89).

## Results

### Patients’ Demographics, Socioeconomic Status, and Clinical Data

**Fig. 1:**
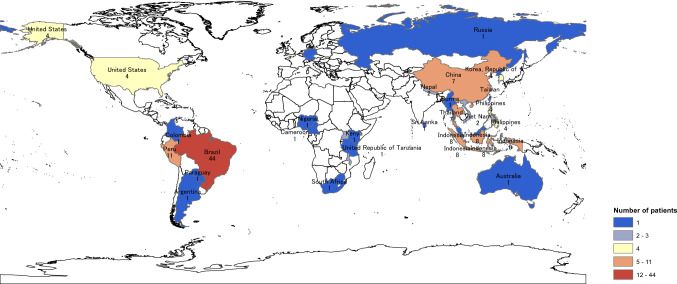
Map of the home countries of the foreign patients in the study sample. Each number represents the number of patients from the country

Of the 931 registered patients, 114 were foreign-born; among them, 44 patients were from Brazil, followed by 11 from Peru and 8 from Indonesia (Fig. 1). A total of 20 patients became lost to follow-up. Patients’ demographics and socioeconomic status by lost-to-follow-up status are presented in Table 1.

**Table 1:** Sociodemographic characteristics of the participants

	Foreign patients		*p*	Japanese patients
	Lost to follow-up			
	Yes (n = 20)	No (n = 94)		n = 817
Age at initial visit, median [IQR]	32 [29–37]	36 [31–47]	0.052	38 [30.9–46.3]
Years of residence in Japan, median [IQR]	10 [3–19]	10 [5–20]	0.49	NA
Sex, n (%)				
Male	17 (85.0)	72 (76.6)	0.41	787 (96.3)
Female	3 (15.0)	22 (23.4)		30 (3.7)
Sexual orientation, n (%)				
Homosexual	5 (25.0)	22 (23.6)	0.47	377 (46.2)
Bisexual	5 (25.0)	12 (12.9)		257 (31.5)
Heterosexual	9 (45.0)	54 (58.6)		152 (18.6)
Other/unknown	1 (5.0)	5 (5.4)		30 (3.7)
At initial visit, n (%)				
Living with family	12 (60.0)	57 (60.6)	0.91	444 (54.4)
Insured	13 (65.0)	85 (90.4)	0.01	784 (96.0)
Employed	11 (55.0)	58 (61.7)	0.35	622 (76.1)
Receiving social welfare	1 (5.0)	7 (7.5)	0.2	411 (5.0)
Receiving disability certificates	4 (20.0)	20 (21.3)	0.9	120 (14.7)
Receiving ART	5 (25.0)	30 (31.9)	0.54	138 (16.9)
AIDS	5 (20.0)	35 (35.1)	0.36	289 (35.4)
AiMIS use n (%)				
Once or more	11 (55.0)	38 (40.4)	0.23	NA
Distance (km) from NMC, median [IQR]	17.6 [3.2–32.0]	16.6 [7.6–31.8]	0.52	NA

*IQR* interquartile range, *ART* anti-retroviral therapy, *AIDS* acquired immuno-deficiency syndrome, *AiMIS* Aichi Medical Interpretation System, *NMC* Nagoya Medical Center, *NA* not applicable

The foreign patients’ median age at initial visits was 36 years for those non-lost-to-follow-up and 32 years for those lost to follow-up (*p* = 0.052). The median years of residence in Japan was 10 years for both groups (*p* = 0.49). The proportion of men was 76.6% in the non-lost-to-follow-up group and 85.0% in the lost-to-follow-up group (*p* = 0.41). A total of 58.6% of patients in the non-lost-to-follow-up group and 45.0% in the lost-to-follow-up group were heterosexual (*p* = 0.47). At the initial visit, 50.0% of patients in the lost-to-follow-up group and 60.6% of patients in the non-lost-to-follow-up group were living with their families (*p* = 0.91). Of patients in the non-lost-to-follow-up group, 90.4% have health insurance as compared to 65.0% in the lost-to-follow-up group, indicating a significant difference (*p* = 0.01). A total of 61.7% of patients in the non-lost-to-follow-up group and 55.0% in lost-to-follow-up group were employed (*p* = 0.35). At the initial visit, anti-retroviral therapy (ART) was introduced to 31.9% of patients in the non-lost-to-follow-up group and to 25.0% of patients in lost-to-follow-up group (*p* = 0.54). Further, 35.1% of non-lost-to-follow-up group patients and 20.0% of lost-to-follow-up group had AIDS (*p* = 0.36).

The Japanese patients’ median age at initial visits was 38 years. Men comprise 96.3% of the sample, and women, 3.7%. A total of 18.6% of Japanese patients were heterosexual. At the initial visit, 54.4% of Japanese patients were living with their families. Of the Japanese patients, 96.0% had health insurance and 76.1% were employed. At the initial visit, ART was introduced to 16.9% of the Japanese patients, and 35.4% of the Japanese patients had AIDS.

### AiMIS Use

Among the 114 foreign-born patients, 49 (43.0%) used AiMIS from the start of this service to August 2017. Among the 49 AiMIS users, Portuguese was the language most required for interpreters (n = 30, 61.2%), followed by Spanish (n = 8, 16.3%) and English (n = 6, 12.2%). Patients’ primary native languages are shown in Supplement 3.

The median number of times AiMIS was used by each patient was five (interquartile range [IQR] = 1–11). Among the 49 patients who utilized AiMIS, 13 (26.5%) used it only once.

Among the 90 patients who were followed up at more than 365 days, 22 patients showed treatment failure. There was no significant difference between patients with and without treatment failure with respect to AiMIS (*p* = 0.25) (Supplement 4).

### Attendance Rate

The median follow-up periods among Japanese and foreign-born patients were 1255 days (IQR = 588–1972) and 809.5 days (IQR = 460–1441), respectively.

**Fig. 2:**
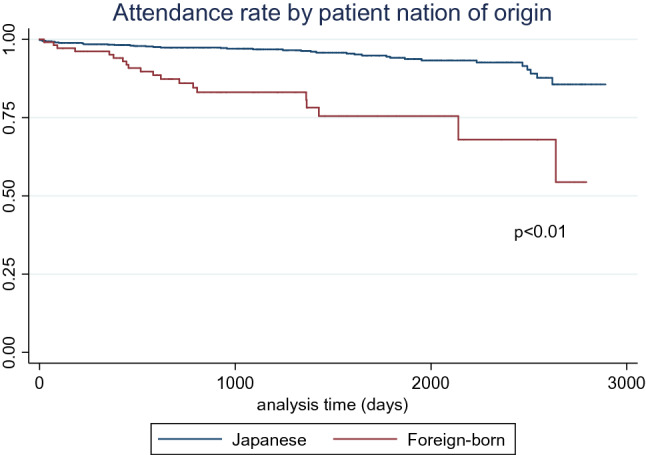
Attendance rate by patient nation of origin

The attendance rate of Japanese patients in our clinic was 98.3%, 97.4%, 96.8%, 95.8%, and 94.1% at Years 1, 2, 3, 4, and 5, respectively. In contrast, the attendance rate of the foreign-born patients after initial visits was 95.1%, 86.0%, 83.1%, 75.5%, and 75.5% at Years 1, 2, 3, 4, and 5, respectively (Fig. 2). The log-rank test indicated that there was a statistically significant difference between Japanese patients’ attendance rate and that of foreign-born patients (*p* < 0.01).

**Fig. 3:**
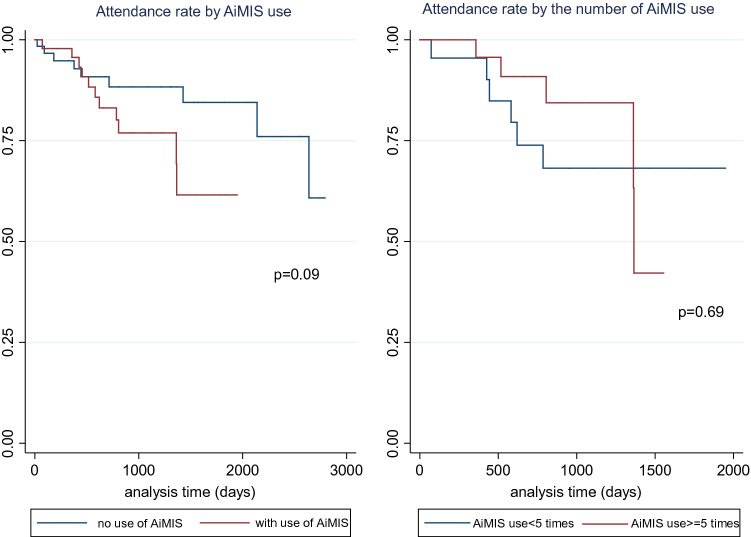
Attendance rate by patients’ AiMIS use and the number of times they used AiMIS

Stratified by the use of AiMIS, the rate of attendance appeared to be higher in the absence of AiMIS use; however, the difference was not significant (*p* = 0.09) (Fig. 3). The rate of attendance seemed to be higher among individuals who used AiMIS more than five times during their follow-up period days until day 1362. The log-rank test, however, showed no significant difference in the attendance rate (*p* = 0.69) (Fig. 3). The attendance rate dropped dramatically after 1362 days. One patient terminated his attendance because he started work outside of the Aichi prefecture, and another did so due to financial problems.

### Determinants of Attendance

Based on the univariate analysis, no factor was related to lost to follow-up (Table 2). We excluded the factor of “Employed” and “Receiving ART” from the multivariate analysis, as the factor “Employed” was strongly correlated to the factor “Receiving social welfare,” and the factor “Receiving ART” also was strongly correlated to the factor “Receiving disability certificates” from a clinical perspective.

**Table 2:** Patient characteristics associated with lost to follow-up

	Univariate analysis	Multivariate analysis
	Odds ratio [95% confidence interval]	Odds ratio [95% confidence interval]
Sex (male)	1.73 [0.46–6.46]	1.71 [0.27–10.90]
Heterosexual	0.59 [0.22–1.56]	0.65 [0.18–2.26]
Living with family	0.7 [0.33–1.50]	0.65 [0.26–1.63]
AIDS at initial visits	1.12 [0.57–2.20]	0.53 [0.15–1.87]
Employed	1.22 [0.61–2.41]	
Insured	0.58 [0.20–1.74]	0.69 [0.27–1.77]
Receiving social welfare	1.89 [0.75–4.75]	4.7 [1.00–22.08]
Receiving disability certificate	0.93 [0.28–3.08]	1.75 [0.44–6.95]
Receiving ART	0.71 [0.24–2.14]	
AiMIS use	1.8 [0.68–4.76]	2.19 [0.66–7.22]
Age at initial visit > 35 years old	0.69 [0.26–1.82]	0.55 [0.17–1.73]
> 10 years of residence in Japan	1.36 [0.50–3.68]	1.83 [0.57–5.86]
Living > 17 km away from NMC	1.11 [0.41–3.00]	1.3 [0.39–4.29]

*NMC* Nagoya Medical Center

Based on the multivariate analysis, the risk of lost to follow-up for males (odds ratio 1.71; 95% CI [0.27, 10.90]; *p* = 0.57) and heterosexual patients (odds ratio 0.65; 95% CI [0.18, 2.26]; *p* = 0.50) did not differ significantly from that of their counterparts (Table 2). Among the socioeconomic factors, no factor was significantly associated with attendance. Insurance was not significantly associated with attendance, after adjusting for patients’ demographics, other socioeconomic factors, and clinical status.

## Discussion

The results showed a significant difference in the attendance rates between Japanese and foreign-born patients. The use of medical interpreters, however, did not significantly affect attendance in this study population.

Our data showed that the attendance rate of the foreign-born population in our HIV clinic at 5 years was 75.5%, which was lower than that of the Japanese patients at that point in time (94.1%). This rate, however, was not necessarily low as compared with other settings. For example, Diaz et al. reported that 13.7% of HIV patients did not attend medical appointments regularly [7]. They also mentioned that one of the factors related to non-regular visit was birthplace, such as Sub-Saharan Africa or Latin-America, indicating that differences between languages and cultures are barriers for patients to engage in regular attendance. In Nepal, only 32.6% of HIV-positive individuals were found to regularly visit a clinic [8]. Regular attendance was promoted by family support, participation in support programs, and knowledge of ART benefits. Regular attendance was hindered, however, by more extended commutes to the clinic and self-rated health status of very good, good, or fair. In our study, AIDS status at the initial visit was not associated with regular attendance. Moreover, the distribution of foreign-born patients’ residences was not different between the non-regular and regular attendance groups. This result may be due to the developed Japanese infrastructure, which facilitates patients visits to hospitals across long distances.

Although we hypothesized that the use of AiMIS would have a positive impact on the attendance of foreign-born PLWH to our clinic, the attendance rates did not show a significant difference when compared by AiMIS use and number of times that AiMIS was used. Therefore, it was assumed that foreign-born patients who did not use AiMIS (1) were able to speak Japanese without an interpreter, (2) had disclosed their HIV infection to family or friends who were able to act as interpreters, or (3) refused to use a medical interpreter because they were afraid that the interpreter could breach confidentiality. One study reported that the disclosure rate of HIV was explicitly lower than that of other chronic diseases, such as viral hepatitis or diabetes mellitus [9]. Therefore, the impact of medical interpreters for patients of other chronic diseases might be more significant than for HIV patients. It also can be assumed that patients who used AiMIS fewer than five times were required to have a medical interpreter based not on their request but on that of the healthcare worker. That is, healthcare workers thought that the patients were not proficient enough in Japanese.

The impact of the use of medical interpreters on HIV-1 treatment was not significant. In regard to this finding, we were able to conclude only that the language barrier was not the only reason for the lost-to-follow-up status among foreign born patients. There might be some other reasons that limited their attendance.

Patient factors did not have an impact on being lost to follow-up when adjusted by multivariate analysis. Nevertheless, “Receiving social welfare” was a significant factor; if the number of participants had been larger, it may have been a factor that had an impact on being lost to follow-up. Thus, further study is warranted.

Our study has some limitations. First, the number of foreign-born nationals was small. Therefore, the lost to follow-up in terms of attendance strongly affected the attendance rate curve when analyzing the impact of AiMIS use. Second, it is impossible to ascertain the reasons that the patients who were lost to follow-up terminated their visits. Thus, we had to assume the reasons for non-attendance. If we could identify the reasons, we would offer assistance with patients’ issues so that they could remain connected to healthcare. Third, our study sample was geographically limited and had different racial characteristics from those of Japan as a whole. The Aichi prefecture, where our facility is located, has approximately 200,000 foreign-born inhabitants, which is the second largest foreign-born population in Japan [10]. Due to manufacturing industries, nearly half work for the factories related to these sectors [11]. A majority are from Brazil, although Chinese are the largest group of residents from foreign nations in Japan [10]. Therefore, if the same analyses were conducted in a different area, the results might differ. Fourth, we analyzed patients who initially visited our clinic from 2009 to 2016, although the AiMIS service did not start until 2012. To minimize selection bias both for foreign-born and Japanese patients, we conducted an analysis that began with 2009, when electronic medical records were introduced into our clinic. Finally, we did not evaluate each patient’s Japanese proficiency objectively. Instead, a subjective assessment of patients’ Japanese speaking ability was made by nurses in our clinic (Supplement 5). According to the data, there was not a significant difference in the Japanese speaking ability between the lost-to-follow-up and non-lost-to-follow-up groups (*p* = 0.70). In the analysis of determinants of attendance, the data were not included due to not being measured objectively. Thus, we are not able to exclude the possibility that there might be a significant difference in the rate of regular attendance according to AiMIS use if we excluded from the analysis the patients who could speak Japanese very well.

Thus, we can conclude that the intervention of medical interpreters did not improve the rate of attendance to our clinic. Hence, to improve the attendance rate of foreign-born patients, not only medical interpreter services, but also comprehensive social support and an employment environment that facilitates receiving healthcare are needed. That is, in Japan, services provided by AiMIS should be combined with administrative and social services.

## Contribution to the Literature

To our knowledge, this study is the first to examine the regular attendance rate of PLWH in Japan, using Kaplan–Meier curves, tracking each patient’s attendance at follow-up visits. This analysis demonstrated significantly different attendance rates between Japanese and foreign-born patients. Further, the use of medical interpreters alone in the clinical setting did not improve the regular attendance rates of foreign-born HIV patients, suggesting that it is necessary to provide not only language support but also comprehensive social support for foreign-born HIV patients.

## Electronic supplementary material

Below is the link to the electronic supplementary material.

Supplementary file1 (DOCX 84 kb)
