# Supplementary material for: Factors Associated with Attrition: Analysis of an HIV Clinic in Japan
Source: J Immigr Minor Health. 2020 Feb 18;23(2):250–6. doi: 10.1007/s10903-020-00982-y (PMC7914243; doi:10.1007/s10903-020-00982-y)
Supplement: Supplementary file 1 — Supplementary file1 (DOCX 84 kb) [file 10903_2020_982_MOESM1_ESM.docx]

**Factors Associated with Attrition: Analysis of an HIV Clinic in Japan**

Chieko Hashiba^1^, Mayumi Imahashi^2^, Junji Imamura^2^, Masashi Nakahata^2^, Ayumi Kogure^2^, Hideto Takahashi^3^, Yoshiyuki Yokomaku^2^

^1^Nagoya Medical Center, Department of Nursing, Nagoya, Japan

^2^Nagoya Medical Center, Department of Infectious Diseases, Nagoya, Japan

^3^National Institute of Public Health, Saitama, Japan

**Online Supplemental Information**

**Definition of terms**

*Social welfare*

The public assistance system for the purpose of supporting minimum standard of living. This includes livelihood, housing, education, medical, long-term care, maternity, and occupational assistance.

<https://www.mhlw.go.jp/english/topics/social_welfare/dl/outline_of_the_public_assistance_system_20101004.pdf>

*Disability certificates*

Medical system to reduce the amount of copayment of medical costs that concern medical services for persons with disabilities. Usually, people who live with HIV in Japan receive these certificates before starting anti-retroviral therapy.

<https://www.mhlw.go.jp/english/wp/wp-hw6/dl/09e.pdf>

**Level of measurement of each variable used in the analysis**

Each variable’s level of measurement is shown below.

[Supplement 1]

**Patients’ native primary language**

There were 18 native primary languages of patients.

[Supplement 2]

The language most required of interpreters was Portuguese (38.6%), followed by Spanish (12.3%) and English (12.3%).

**Spatial analysis**

To analyze distances between patients’ residency address and our hospital, we used Geographic Information System (GIS) analysis. First, we geocoded patients’ residency addresses and our hospital address, using ArcGIS Online (ESRI Corp., Redlands, California, USA). Second, these data points were merged with a layer file of the hospital. Third, we measured the distances between the hospital and each point. We compared the distances between the lost-to-follow-up group and non-lost-to-follow-up group, using the Mann-Whitney U test, as the data on distances were not normally distributed. This analysis was conducted using the ArcGIS Desktop ver. 10.5 (ESRI Corp., Redlands, California, USA).

[Supplement 3]

A total of 107 patients out of 114 provided their residence addresses, and these were successfully geocoded (Supplement 3). For the patients in the lost-to-follow-up group, the median distance from the hospital was 17.6km (IQR: 3.2-32.0), while, for the non-lost-to- follow-up group, the distance was 16.6km (IQR: 7.6-31.8). There was no significant difference in the distances between the hospital and patients’ residence (*p =* .67) (Table 1).

**Treatment failure and AiMIS use**

We examined the treatment failure rate among the patients with whom we followed up for more than 365 days (n = 90). We defined treatment failure as having an HIV-1 viral load of more than 200 copies/ml. Among the 90 followed-up patients, 22 showed treatment failure at least once during the entire follow-up period. Among these 22 patients, 9 (40.9%) used AiMIS. There was no statistical difference, however, between patients with and without treatment failure (*p =* .48). Of those who had used AiMIS in the past year, 7 patients showed viral treatment failure (5 in the AiMIS-use group and 2 in the non-AiMIS-use group). There was no significant difference, however, between patients with and without treatment failure in terms of AiMIS (*p =* .25)

[Supplement 4]

**Subjective assessment of patients’ Japanese speaking ability**

Nurses in our clinic assessed patients’ Japanese speaking ability during their initial visits. Subjective evaluation was conducted for 76.3% of foreign-born patients. Of the 114 foreign-born patients, 66 (57.9%) were evaluated as they were able to communicate orally with nurses. There was no significant difference in patients’ Japanese speaking ability between the lost-to-follow-up and non-lost-to-follow-up groups (*p = .70*).

[Supplement 5]

Supplement 1. Level of measurement of each variable used in the analysis

| Variable | Level |
| --- | --- |
| Age at initial visit | Continuous |
| Years of residence in Japan, | Continuous |
| Sex | Nominal |
| Sexual orientation | Nominal |
| At initial visit |  |
| Living with family | Nominal |
| Insured | Nominal |
| Employed | Nominal |
| Receiving social welfare | Nominal |
| Receiving disability certificates | Nominal |
| Receiving ART | Nominal |
| AIDS | Nominal |
| AiMIS use |  |
| Once or more | Nominal |
| Distance from NMC | Continuous |

*Notes.*

ART: anti-retroviral therapy, AIDS: acquired immuno-deficiency syndrome, AiMIS: Aichi Medical Interpretation System, NMC: Nagoya Medical Center

Supplement 2. Patients native primary language

| Language | n (%) |
| --- | --- |
| Portuguese | 44 (38.6) |
| Spanish | 14 (12.3) |
| English | 14 (12.3) |
| Indonesian | 8 (7.0) |
| Thai | 7 (6.1) |
| Chinese | 7 (6.1) |
| Korean | 5 (4.4) |
| Ugandan | 3 (2.7) |
| Tagalog | 2 (1.8) |
| Vietnamese | 2 (1.8) |
| Tanzanian | 1 (0.9) |
| Nepali | 1 (0.9) |
| Hindi | 1 (0.9) |
| French | 1 (0.9) |
| Malay | 1 (0.9) |
| Myanmar | 1 (0.9) |
| Russian | 1 (0.9) |
| Japanese | 1 (0.9) |


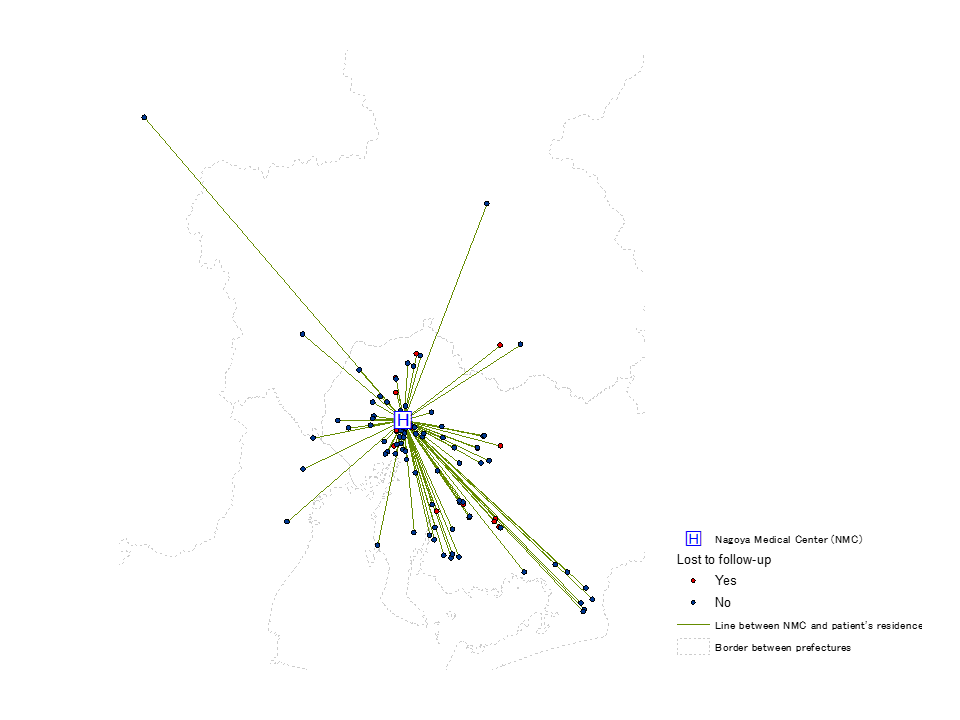


Supplement 3. Distance between patients’ residences and Nagoya Medical Center

Supplement 4. Viral Treatment Failure and AiMIS Use

|  |  | AiMIS use | |  |
| --- | --- | --- | --- | --- |
| Viral treatment failure occurred at least once within | | No | Yes | Total |
| Whole observed days | 1 year of the last  observed day |  |  |  |
| Occurred | Occurred | 2 | 5 | 7 |
|  | Did not occur | 11 | 4 | 15 |
| Total | | **13** | **9** | **22** |
| Did not occur | | **34** | **34** | **68** |
| Total | | **47** | **43** | **90** |

The first Total refers to the sum of the values in two rows above, whereas the second total refers to the first total plus the row below the first total.

Supplement 5. Subjective assessment of patients’ Japanese speaking ability

|  | Lost to follow-up | |  |
| --- | --- | --- | --- |
| Oral communication (n (%)) | Yes (n = 20) | No (n = 94) | *p* |
|  |  |  |  |
| Not available | 4 (20.0) | 17 (18.1) | 0.70 |
| Available | 10 (50.0) | 56 (59.6) |  |
| Not evaluated | 6 (30.0) | 21 (22.3) |  |
